# Is there a future for ^43^Ca nuclear magnetic resonance in cement science?†

**DOI:** 10.1039/d5cp00491h

**Published:** 2025-04-09

**Authors:** Ziga Casar, Davide Tisi, Samuel J. Page, H. Chris Greenwell, Franco Zunino

**Affiliations:** a Department of Chemistry, Durham University South Road Durham DH1 3LE UK casar.ziga@outlook.com; b Department of Civil and Environmental Engineering, Princeton University Princeton NJ 08544 USA; c Laboratory of Computational Science and Modeling, Institut des Matériaux, Ecole Polytechnique Fédérale de Lausanne (EPFL) 1015 Lausanne Switzerland; d Department of Civil and Environmental Engineering, University of California at Berkeley USA zunino@berkeley.edu

## Abstract

Calcium and silicon are critical components of cement. While ^29^Si nuclear magnetic resonance (NMR) is widely used in cement science, ^43^Ca NMR has received comparatively less attention given the experimental challenges associated with it. To investigate the potential of ^43^Ca NMR in cement research, a density functional theory study was carried out. The study focused on distinct calcium sites within the calcium silicate hydrate (C–S–H) structure. Four unique calcium sites were identified, each predicted to display distinct ^43^Ca chemical shifts due to differences in their local environments. These findings were used to generate theoretical ^43^Ca NMR spectra for C–S–H. Furthermore, theoretical ^43^Ca NMR spectra for the hydration reaction of triclinic tricalcium silicate were developed, illustrating the potential of ^43^Ca NMR for tracking the hydration process in multiphase systems.

## Abstract 2

This article provides a theoretical investigation into ^43^Ca NMR for cementitious systems.

## Graphical abstract

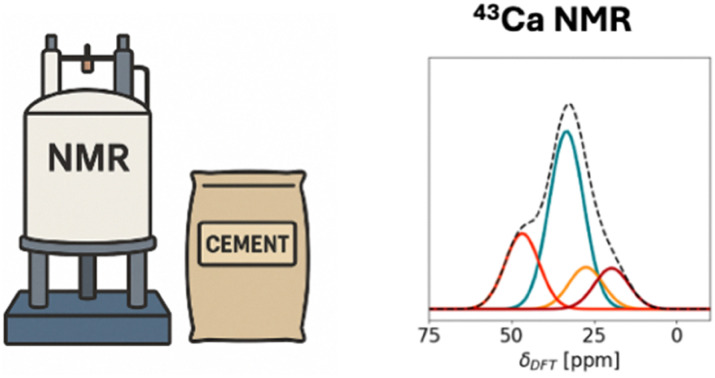

## 1 Introduction

With an annual production exceeding 5000 Mt and a projected demand that continues to rise steadily, cement stands out as the most extensively manufactured material on the planet.^1^ Cement-based materials alone represent over 30% of the global material production output and are responsible for emitting approximately 8% of anthropogenic CO_2_ emissions.^1^ The pressing need to develop cement with reduced carbon footprint is coupled with the increasing utilization of supplementary cementitious materials (SCMs).^2^ However, the use of SCMs results in lower initial reactivity and therefore lower early age compressive strength. This limitation serves as a compelling impetus for more foundational research in the area of cement hydration.

The process of cement hydration involves a complex interplay of coupled reactions among various mineral phases, each characterized by distinct kinetics and mechanisms.^3^ Within the initial 24 hours of hydration, cement undergoes a significant transformation from a fluid, colloidal suspension of solid particles, to a solid, porous medium exhibiting measurable engineering properties such as strength and durability. A substantial portion of alite (C_3_S in cement notation, 70–80%) and tricalcium aluminate (C_3_A, 80–100%) react within this timeframe,^4–6^ resulting in the precipitation of key hydration products including calcium silicate hydrates (C–S–H), its aluminium incorporating counterpart (C–A–S–H), calcium hydroxide (CH), and ettringite (C_6_A$_3_H_32_).

C–(A)–S–H is the main hydration product of Portland cement (most common type of cement), accounting for about 50–60% of the total volume of hydrates.^7^ C–(A)–S–H exhibits a layered calcium–silicate structure resembling the natural mineral tobermorite.^8^ In contrast to the tobermorite minerals the silicate chain structures is defective (missing silicates) and the interlayer space contains a higher amount of ions, most notably Ca^2+^ and OH^−^.^8,9^ These features give C–(A)–S–H its nanocrystalline character, as observed by X-ray diffraction, where characteristic reflections are seen and are assigned to repeating distances in the calcium–silicate mainlayer.^10–12^

Most of the fundamental knowledge about the C–(A)–S–H structure derives from the coupling of solid-state nuclear magnetic resonance (NMR, mainly ^29^Si, ^27^Al and ^1^H) and atomistic simulations.^13–17^ The Ca/Si ratio in C–(A)–S–H (1.4–2.0) is substantially higher than in tobermorite (0.83) due to incorporation of Ca^2+^ in the interlayer^9,17^ and Ca^2+^ adsorption at the surface.^18^ In low-carbon cements, the incorporation of Al in C–(A)–S–H is of particular interest due to the presence of alumina-bearing SCMs (fly ash, calcined clays, natural pozzolans) in the cement mixture. It has been shown that Al is incorporated in vacant silicate-bridging sites, stabilized by hydroxyl groups and Ca^2+^ in the interlayer.^19,20^

Efforts to enhance signal detection in NMR, such as cross-polarization (CP) and dynamic nuclear polarization (DNP), have been extensively employed to improve the signal-to-noise ratio and address the analytical challenges associated with analyzing C–(A)–S–H, which hosts a plethora of coexisting Si and Al species.^17,19,21^ CP involves transferring polarization from protons to the nucleus of interest,^22–24^ while DNP facilitates polarization transfer from electrons to nuclear spins.^25,26^

While Si defines the backbone of C–(A)–S–H, Ca constitutes most of the mainlayer and dominates the interlayer and surface interactions in C–(A)–S–H.^9,18^ However, the fate of Ca in C–(A)–S–H upon hydration is considerably less understood than Si and Al, largely explained by the challenge to measure Ca with NMR.

This difficulty in studying Ca^2+^ with NMR arises due to the combined contribution of the extremely low natural abundance of the Ca active nuclei (^43^Ca, 0.145% natural abundance, which contrasts with the 100% of ^27^Al and 4.7% of ^29^Si) and its quadrupolar nature (spin −7/2). Furthermore, a low frequency probe is needed (frequency at 600 MHz/14.1T of 40.368 MHz) which hinders the ability to experimentally access this nucleus in facilities where only common broadband probes are available. On the other hand, 60–70% by mass of a conventional clinker corresponds to CaO, highlighting the pivotal nature of this element in cement science.

In their pioneering work, Bowers and Kirkpatrick,^27^ showed that ^43^Ca MAS NMR spectra with reasonable signal-to-noise ratios for tobermorite and jennite (both calcium silicate hydrate minerals) with natural abundance can be obtained. In particular they observed that differences between 6-fold and 7-fold coordinated Ca are large enough to be entirely resolved by ^43^Ca NMR. Afterwards, Moudrakovski *et al.*^28^ carried out a combined natural abundance ^43^Ca NMR and density functional theory (DFT) study. They investigated anhydrous phases (C_3_S, β-C_2_S, C_3_A), hydrated C_3_S, Ca(OH)_2_, tobermorite 11 Å, and low Ca/Si (below 1.5) synthetic C–S–H. Their results suggest significant differences in the ^43^Ca NMR spectra of the observed phases due to differences in the local Ca environment. Further, due to the similarities between the ^43^Ca NMR spectra of tobermorite 11 Å and C–S–H they further confirmed the validity of using layered tobermorite models as model structures for C–S–H. Finally, MacDonald *et al.*^29^ used ^43^Ca NMR to show that poly(ethylene-vinyl acetate) (PEVAc) admixtures in white cement induce modest structural changes, while describing the formation of low-coordination Ca sites upon interaction with PEVAc.

Past measuring attempts at natural abundance of ^43^Ca show promise,^27–29^ but are, however, limited by the length and resolution (signal-to-noise ratio) of the spectra obtained. While some reviews are less optimistic about the prospects of ^43^Ca NMR in cement science,^30^ it is expected that the situation might improve in the future as larger fields (>20 T) become readily available and better routines for signal enhancement are developed. For example, Shimoda *et al.*^31^ studied calcium silicate glasses with multiple-quantum magic angle spinning (MQMAS) NMR that they synthesized from ^43^Ca-enriched CaCO_3_. Although they obtained highly resolved 5QMAS spectra at 16.4 T for Mg and Al incorporating calcium silicate glasses, 7QMAS at 21.8 T was needed to show the 8-, 7- and 6-fold coordinated Ca environments in CaSiO_3_. Other methods, which can offer high-resolution spectra for half-integer spins are double rotation (DOR), dynamic angle spinning (DAS), and ^43^Ca–^1^H rotational-echo double resonance (REDOR).^31–33^

Further, due to the advances in atomistic modeling techniques over the past 10 years^34,35^ a better atomic-level understanding of the anhydrous and hydrated phases of Portland cement has been obtained. Particular advances were achieved in elucidating the C–S–H structure,^17,18,34^ where it was shown that combining DFT predicted chemical shieldings with experimentally measured (^1^H, ^27^Al and ^29^Si) NMR spectra can be successfully used to gain atomic-level insight into the C–S–H structure.^19,21^

Since all major anhydrous and hydrated phases of Portland cement contain calcium we raise a question: is there a future for ^43^Ca NMR in cement research? To answer this question a theoretical study of DFT predicted ^43^Ca NMR chemical shifts was carried out. By using more accurate models we explore how ^43^Ca NMR could further advance our understanding of the C–S–H structure and be of value in gaining atomic-level insights into the cement hydration process.

## 2 Methods

### 2.1 Calcium silicate hydrate model structures

Nine unique C–S–H bulk structures containing 1 or 2 defective tobermorite 14 Å unit cells were generated with the brick model methodology from Kunhi Mohamed *et al.*^9,36^ The typical C–S–H defects (missing Q^2b^ silicate, addition of Ca^2+^ and OH^−^ ions in the interlayer) were accounted for.^9^ Through these defects C–S–H structures with Ca/Si ratios between 1.4 and 1.8 were generated. Afterwards, the water and ions in the C–S–H interlayer of the unique structures were randomly shuffled. Through this 77 different starting configurations were obtained. Further, the interlayer space of 3 unique structures was expanded through addition of water molecules to create two C–S–H (001) basal surfaces. The water slab between the surfaces was approximately 2 nm. Again, the water and ions were randomly shuffled to create 32 starting configurations for the C–S–H surface. Finally, 16 starting configurations with one Ca^2+^ and two OH^−^ ions in a water box were generated. All structures are part of the ESI† section.

Before carrying out the density functional theory (DFT) calculations (see next section) each structure was pre-relaxed through energy minimization. LAMMPS^37^ in conjunction with the EricaFF2 force field^38,39^ was used. Details of the energy minimization can be found elsewhere.^21^

In addition to the generated structures, 98 zinc-doped C–S–H bulk structures from Morales *et al.*^21^ were analyzed as well. Morales *et al.* studied the ^29^Si DFT predicted chemical shieldings. However, when calculating the chemical shieldings, they are calculated for each atom in the system, and therefore the Ca chemical shieldings were reported as part of the ESI.†

Each final C–S–H structure (after DFT) was visualized and inspected. For each Ca in the structure its location (site), Ca–O coordination number, average Ca–O bond distance, the calcium polyhedra volume, and the calculated chemical shielding were determined and stored in a CSV file. The cutoff distance for Ca–O was 3.15 Å. The CSV file is given as part of the ESI.†

### 2.2 Density functional theory

DFT is one of the most widely used tools in computational chemistry thanks to its accuracy and affordable computational efficiency. This method has became a standard tool in material science due to its application in predicting the structures and properties of materials, as well as the reactivity of different phases.^35,40^

For every compound we optimized the atomic positions and lattice parameters using DFT self consistent calculation based on the generalized gradient approximation (GGA), using the Perdew–Burke–Ernzerhow (PBE)^41^ functional and Grimme D2 dispersion corrections,^42,43^ which are used to obtain reliable relaxed structure. Projected augmented wave scalar relativistic pseudopotentials were used from PSlibrary version 1.0.0 were used.^44,45^ Semicore s and p electrons were included for calcium, and nonlinear core correction was applied for heavy atoms. Wave function and charge density energy cutoffs were set to 80 and 320 Ry. A Monkhorst–Pack grid of *k*-points corresponding to a maximum spacing of 0.05 Å^−1^ was used.^46^ Following the geometry optimization, a single-point computation was carried out with the same parameters, and chemical shieldings were computed using the GIPAW method.^47,48^ The computational setup and choice of DFT functional represent a good compromise between computational efficiency and accuracy, and have been shown to accurately reproduce the characteristics of similar materials.^19,21^

**Fig. 1:**
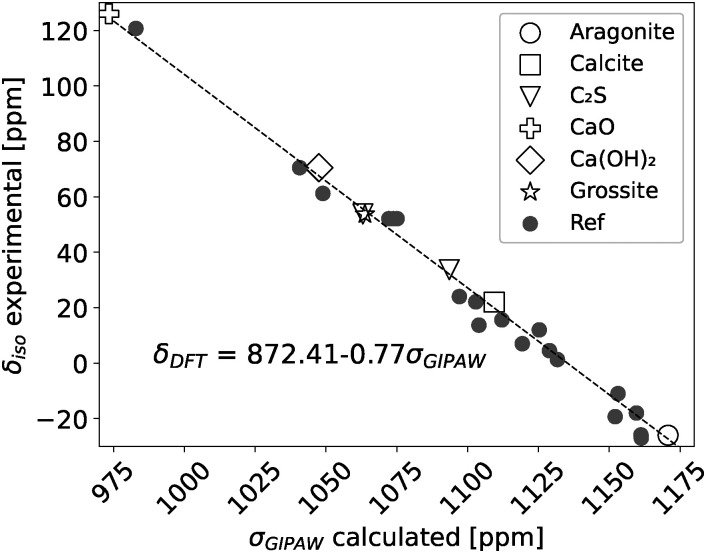
Experimental ^43^Ca NMR chemical shifts (*δ*iso) and DFT calculated chemical shieldings of references structures which were used to define the scaling factor (*δ*_DFT_). Reference DFT calculations were taken from Moudrakovski *et al.*^28^ Experimental ^43^Ca NMR shifts are from Laurencin *et al.*^55^

To correlate the NMR measured chemical shift (*δ*iso) with the DFT calculated chemical shielding (*σ*_GIPAW_) a reference set of crystal structures with well known NMR chemical shifts was chosen. The reference set in this study for correlating the ^43^Ca NMR shifts with the calculated chemical shieldings consisted of aragonite,^49^ calcite,^50^ β-dicalcium silicate,^51^ calcium oxide,^52^ calcium hydroxide^53^ and grossite.^54^ The calculated chemical shieldings (*σ*_GIPAW_) of the reference structures with their corresponding experimental chemical shift (*δ*_iso_)^55^ are shown in Fig. 1. Fig. 1 includes the DFT predicted chemical shieldings from Moudrakovski *et al.*,^28^ which are in good agreement with the here reported shieldings. The linear regression fit yields then the scaling factor, *δ*_DFT_ = 872.41 − 0.77*σ*_GIPAW_, which was used to convert the chemical shieldings to chemical shifts for the investigated (C–S–H) structures.

The ^43^Ca chemical shifts of the different Ca sites are presented as the mean value. The ^43^Ca NMR spectrum of each calcium site was modeled as a normal distribution with the mean value (as *μ*) defining the center of the distribution and the typical full width at half maximum (FWHM as *σ*) controlling the width. FWHM was chosen as 5.2 ppm. This is the FWHM of 60% enriched calcium benzoate trihydrate ^43^Ca MAS NMR spectra.^56^ Comparable FWHM are reported for natural abundance ^43^Ca MAS NMR spectra of calcium carbonate.^32^

All DFT computations were carried out with Quantum ESPRESSO v7.2 and its implementation of the plane-wave DFT method.^57–59^ The input and output files are part of the ESI.†

### 2.3 Hydration kinetics thermodynamic modeling

The Parrot and Killoh model^60^ modified by Lothenbach *et al.*^61^ was used to compute the degree of hydration (DoH) evolution of a C_3_S paste over time with a water-to-solids ratio of 0.5. The output of the model was then taken as input (GEMS version 3 software^62^), coupled with the CEMDATA18 thermodynamic database^63^ to compute the phase (C–S–H, CH) at each time step. While several assumptions are made in this calculation, the model provides a rough overview of the relative proportions of anhydrous C_3_S and hydrates at different DoH, which was then used to generate ^43^Ca NMR spectra of these systems.

## 3 Results and discussions

The results section is structured as follows. First, 4 unique Ca sites are identified. The sites are identified by their local environment which stems from their location in the C–S–H nanofoil. This local environment is defined by the Ca connectivity, such as the coordination number and oxygen type, which consequently impacts the symmetry of the nuclei and therefore the broadness of the ^43^Ca NMR shift. According to the DFT predicted chemical shifts of the model C–S–H structures a distinct isotropic chemical shift is assigned to each of the 4 unique Ca sites in C–S–H. All chemical shielding tensors are provided as part of the ESI.† Then, existing experimental measurements of different C–S–H structures are used to build C–S–H nanofoil models. For these nanofoil models the proportion of the individual Ca sites is then calculated and used to predict ^43^Ca NMR spectra of C–S–H. Finally, ^43^Ca NMR spectra for different C_3_S hydration rates are predicted and compared.

### 3.1 Calcium sites in calcium silicate hydrate

**Fig. 2:**
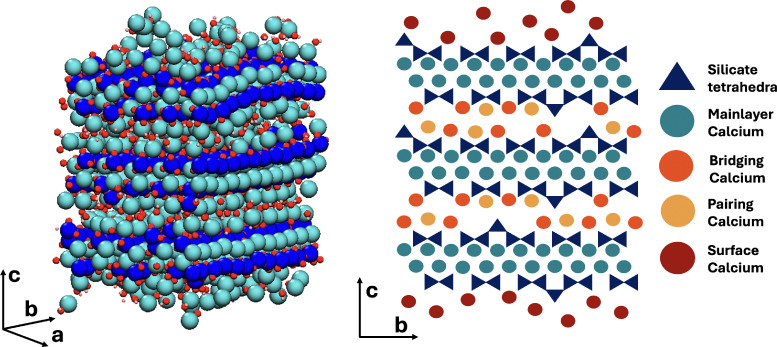
Atomistic model of the C–S–H nanofoil as proposed by Casar *et al.*^18^ (left) and a 2D schematic representation of the C–S–H nanofoil with the distinguished calcium sites (right).

The atomistic model of the C–S–H nanofoil as proposed by Casar *et al.*^18^ and a 2D schematic of the 3 layer, 2 interlayer thick C–S–H nanofoil are shown in Fig. 2. From the current understanding of the C–S–H structure, 4 unique Ca sites can be identified: mainlayer, bridging, pairing, and surface. Each of these sites reflects a different structural environment of the Ca atom and therefore is expected to have a distinct ^43^Ca chemical shift.^28^ However, a lack of symmetry is expected, and therefore broadening by the second-order quadrupolar interactions is anticipated.^28,55^ The possible Ca sites in the C–S–H nanofoil are shown in Fig. 2 and are then described in detail below.

#### 3.1.1 Mainlayer calcium site

X-ray diffraction patterns of synthetic C–S–H show a remarkable resemblance with the tobermorite 14 Å structure and can be fitted with its monoclinic *B11b* cell with a high accuracy.^10,64^ Although it is known from ^29^Si NMR that the C–S–H silicate chain structure is highly defective (missing bridging Q^2b^ sites)^8,17^ the resemblance of the X-ray diffraction pattern, including the position of its peaks, suggests very similar if not nearly identical ordering of the mainlayer.

**Fig. 3:**
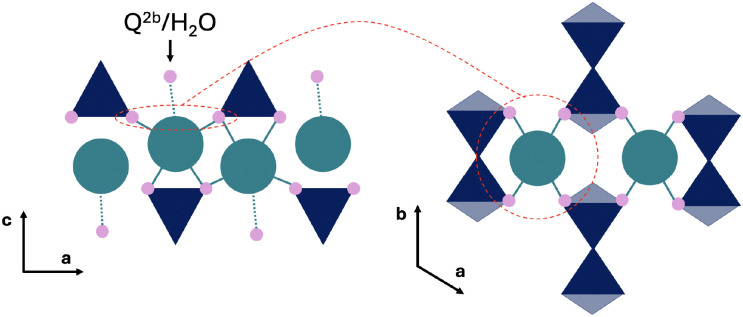
Schematic representation of the mainlayer Ca site. Color legend: dark blue – silicate tetrahedra, turquoise – mainlayer Ca site, pink – Ca connecting oxygen.

Since the mainlayer Ca in tobermorite 14 Å is 7-fold coordinated, a highly similar connectivity is expected in the C–S–H structure. In tobermorite 14 Å six Ca coordinating oxygens are from the in-chain Q^2p^ silicates and the seventh is from the bridging Q^2b^ silicate or a water molecule. Along the *b*-axis direction in tobermorite 14 Å the water and Q^2b^ oxygen interchange.^64^ The 7-fold coordination of mainlayer Ca is shown in Fig. 3.

Molecular dynamics studies showed that a 7-fold Ca coordination number in C–S–H is achieved through an oxygen of the water molecule or a hydroxide ion in the interlayer space.^36^ Since the mainlayer Ca site is located in the well-structured Ca-Si mainlayer, a higher symmetry in comparison to other sites is expected (Fig. 3). Previous studies on synthetic C–S–H suggest that an increase in the Ca/Si ratio promotes structural ordering,^10^ which could result in even higher local symmetry.

The here generated DFT database of chemical shieldings for C–S–H model structures was analyzed for mainlayer Ca sites with an 7-fold coordination whereby only those sites were considered that are coordinated to 6 oxygens of in-chain silicates (Q^1^ or Q^2p^). The seventh oxygen is either from a Q^2b^ silicate, a water molecule, or a hydroxide ion. For this Ca site a mean ^43^Ca chemical shift of 33.38 ppm is predicted (Table 1).

**Table 1:** Ca sites and their Ca–O coordination number (CN), polyhedra volume and mean ^43^Ca chemical shift (*δ*_DFT_) as predicted by DFT. The note refers to the conditions of the Ca–O CN

Ca site	Ca–O CN	Poly. vol. [Å^3^]	*δ*_DFT_ [ppm]	Note
Mainlayer	7.00 ± 0.0	21.43 ± 1.16	33.38	6·O(Si)
Bridging	6.27 ± 0.47	17.82 ± 3.13	46.84	CN 6+; 2·O(Si)
Pairing	7.22 ± 0.46	22.93 ± 3.19	27.50	CN 7+
Surface	7.14 ± 0.36	22.66 ± 2.54	19.72	CN 7+
Solution	7.42 ± 0.50	23.98 ± 3.26	−0.01	CN 7+; only H_2_O

The DFT predicted ^43^Ca chemical shifts for tobermorite 11 Å and 14 Å, which are expected to have the same mainlayer Ca structure as C–S–H,^64,65^ are at 23 and 25 ppm.^28^ However, the ^43^Ca shift shifts to more positive values as the Ca/Si increases due to Ca being deshielded.^28^ The deshielding is due to the seventh coordination stemming from the water molecule or the hydroxide ions instead of the Q^2b^ silicate oxygen. Since the number of Q^2b^ sites decreases with an increase in Ca/Si,^34^ a shift of the mainlayer ^43^Ca shift to more positive values was expected.

#### 3.1.2 Interlayer calcium sites

**Fig. 4:**
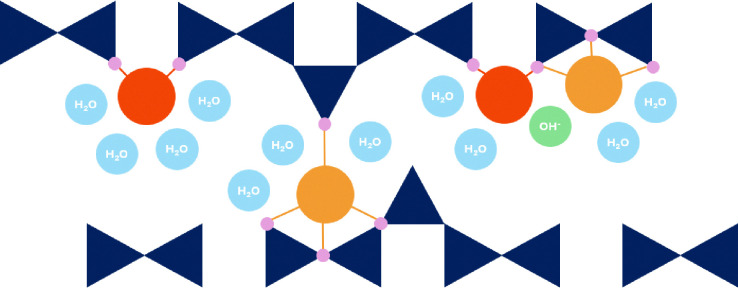
Schematic representation of bridging (red) and pairing (orange) Ca site. Dark blue are the silicate tetrahedra, small blue circles represent water molecules and green is the hydroxide ion (OH^−^).

Assignment of the interlayer Ca sites is still controversial (Fig. 4). However, Ca in the vacant Q^2b^ site (bridging Ca) is reasonably established in the literature,^17,18,66,67^ since it seems to act as a connection bridge between the individual silicate chain units. Indeed, Zhang *et al.*^68^ showed with *ab initio* calculations that Ca electrostatic linking of silicate chains has a smaller energy barrier than the Si–O–Si bond formation.

The second possible Ca site is the pairing Ca, which is positioned on top of the silicate dimer (Q^1^–Q^1^, Q^1^–Q^2p^ or Q^2p^–Q^2p^).^69^ While there is yet no experimental evidence for this Ca site, it is a reasonable assumption since: (1) the typically reported C–S–H interlayer distance (below 1.3 nm)^8^ is too small for a Ca being solely fully hydrated by water and hydroxide ions, and (2) Ca adsorbs, in contrast to the solid–liquid surface, strongly in confined spaces.^70,71^

The main difference between the bridging and pairing Ca is in the coordination, which is known to influence the measured chemical shift.^55^ As the Ca–O coordination number increases the ^43^Ca chemical shift decreases.^72^ Since, in this case, both sites are exposed to an identical interlayer environment, a similar coordination to water and hydroxide ion oxygens can be expected. Therefore the difference arises from the silicate oxygens. Since the bridging Ca connects two Q^1^ silicates it can only coordinate to two silicate oxygens, while the pairing Ca sitting on top of the dimer therefore can coordinate to 3 silicate oxygens. Depending on the interlayer distance, the pairing site can potentially coordinate to a fourth oxygen of the Q^2b^ silicate of across the interlayer silicate chain. This across interlayer connectivity of the Ca pairing site is only possible due to the silicate chains of the tobermorite-like mainlayer being shifted by *b*/2.^64^

Indeed from the DFT relaxed C–S–H bulk structures in this study, the mean Ca–O coordination number of bridging Ca equals 6.04 and 6.42 for the pairing Ca. This finding aligns well with experimentally determined Ca–O coordination numbers of C–S–H. Typical reported values for Ca–O coordination number in C–S–H are between 5.9 and 6.6.^36^ Assuming 7-fold mainlayer Ca, the decrease in the overall Ca–O coordination number can then only be due to lower coordinated Ca in the interlayer.

It is expected that a bridging Ca coordinates to one oxygen less than a pairing Ca. For the bridging site a Ca–O coordination number of 6 or higher was assumed. Simultaneously, the bridging Ca needs to be coordinated to two silicate oxygens. Our DFT database predicts for such a Ca site a ^43^Ca chemical shift of 46.84 ppm. For the pairing Ca the database was screened for pairing sites with a Ca–O coordination of 7 or higher from which at least 2 need to be to silicate oxygens. Such a pairing site is expected to have a ^43^Ca chemical shift of 27.50 ppm.

#### 3.1.3 Surface calcium sites

**Fig. 5:**
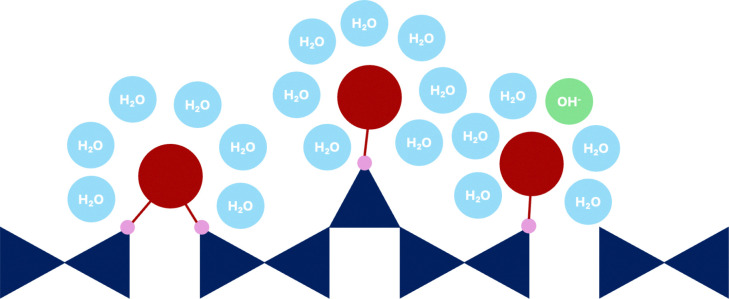
Schematic representation of surface Ca sites (dark red). Dark blue are the silicate tetrahedra, small blue circles are the water molecules, and green is the OH^−^ ion.

To reach a high Ca/Si C–S–H, as in real cementitious systems, C–S–H needs to be precipitated in a high pH, Ca-rich solution.^73^ In such systems, a difference between the Ca concentration in solution after synthesis as predicted by thermodynamic modeling, and experimentally measured is observed,^73^ which suggests high adsorption rates of Ca at the surface.^18,74^ It is expected that Ca sites near the particle surfaces (Fig. 5) will have local symmetries, bond angles, and bond distances that differ from those in the bulk C–S–H and therefore a different chemical shift.^74^

In contrast to the confined interlayer surfaces the Ca ions at the external surface have more space and are expected to fully fill their first hydration shell. A full hydration shell results in a bigger polyhedra volume, more oxygens shielding the Ca nuclei, and therefore lower chemical shifts.^72^ However, similar to the interlayer Ca position a loss in symmetry is expected, and therefore broader NMR peaks.

We define a surface site as an inner-sphere adsorbed Ca which can coordinate to one or two silicate oxygens (Fig. 5).^75^ As expected, the DFT calculated surface Ca sites show the lowest mean *δ*_DFT_ (Table 1). This can be attributed to a higher mean Ca–O coordination number (7.14) and therefore a larger polyhedra volume, and consequently a larger mean Ca–O distance.^55,72,76^ The expected ^43^Ca chemical shift for Ca ions at the interface (inner-sphere adsorbed) is 19.72 ppm. It is observed that with an increased coordination to silicate oxygens O(Si) the mean *δ*_DFT_ shifts to more positive values (16.32 ppm when coordinated to one silicate oxygens and 21.08 ppm when coordinated to two), showing the effect of silicate oxygen shielding onto the Ca nuclei.

The solution Ca site in Table 1 is a fully hydrated Ca^2+^, which does not coordinate to the silicate oxygens of the surface and can be considered as an outer-sphere adsorbed Ca^2+^. For this site, a *δ*_DFT_ of −0.01 ppm is calculated, which corresponds to a fully hydrated Ca^2+^ as in CaCl solution, the reference system for ^43^Ca NMR.^55^

Table 1 summarizes the identified Ca sites. These Ca sites were used in generating the theoretical spectra in the next sections.

### 3.2 ^43^Ca NMR spectrum of calcium silicate hydrate

**Fig. 6:**
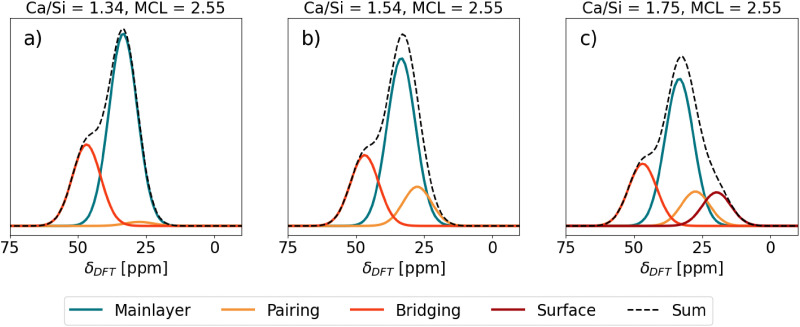
Predicted ^43^Ca NMR spectra of different C–S–H structures. SUM stands for the total contribution of the spectra of the individual Ca sites.

The Ca sites in Table 1 were used in generating theoretical ^43^Ca NMR spectra of C–S–H shown in Fig. 6. Each total spectrum (Sum) is normalized to 1 and the area of the individual Ca site spectra corresponds to the fraction of the total spectrum that the Ca sites occupy in the given C–S–H structure.

First, C–S–H with Ca/Si = 1.34 and mean chain length (MCL) = 2.55 (Fig. 6(a)) corresponds to a typical low Ca/Si C–S–H as might be synthesized from CaO and SiO_2_ without the presence of portlandite.^77^ A C–S–H nanofoil model of 3-layer thickness^18^ with this Ca/Si and MCL results in a structure that has, in addition to Ca in the mainlayer, Ca occupying all vacant Q^2b^ sites. With only the mainlayer (69% of all Ca sites) and bridging Ca site (31% of all Ca sites), the Ca/Si ratio of 1.34 at MCL = 2.55 is achieved. No Ca adsorption at the surface is needed. As seen in Fig. 6(a) the expected ^43^Ca NMR spectrum has a clear peak at 33.5 ppm due to the prevalence of mainlayer ^43^Ca site and a formation of the left shoulder, due to the bridging ^43^Ca site with *δ*_DFT_ = 46.84 ppm.

Second, C–S–H in Fig. 6(b) assumes the same MCL structure and has no Ca^2+^ surface adsorption, which is the same as the C–S–H model in Fig. 6(a). However, the additional assumption is a 0.7 Ca/dimer packing in the interlayer,^9^ which results in 0.35 Ca pairing sites per silicate dimer in the C–S–H interlayer. In such a C–S–H structure roughly one-third of interlayer silicate dimers coordinate with pairing Ca, while approximately three-fourths of bridging sites are occupied by bridging Ca. This additional occupancy of the pairing site is seen as a slight right broadening of the mainlayer Ca peak in Fig. 6(b), due to the pairing site exhibiting a 27.50 ppm chemical shift, which overlaps with the right side of the mainlayer peak. Due to this broadening the mainlayer peak shifts from 33.5 to 33 ppm.

Fig. 6(c) represents the same C–S–H structure as Fig. 6(b), except Ca adsorption at the surface is assumed that increases the Ca/Si ratio to 1.75.^18^ A C–S–H structure with these properties was synthesized by Kumar *et al.*^17^ who used the drop-wise precipitation method. It is seen that due to the surface site (19.72 ppm), a right shoulder of the main peak forms. The position of the main peak remains at 32.5 ppm.

Moudrakovski *et al.*^28^ carried out a natural abundance high field ^43^Ca NMR study where they studied C–S–H with Ca/Si of 0.8, 1.2 and 1.5. The reported experimental ^43^Ca NMR spectra are in good agreement with the here presented theoretical findings. The experimental ^43^Ca NMR peak center of C–S–H with Ca/Si = 0.8 aligns well with the tobermorite 14 Å peak at ∼25 ppm,^28^ while for Ca/Si of 1.2 and 1.5 it is at >30 ppm which agrees with the here reported DFT predicted chemical shift of mainlayer Ca (Table 1 and Fig. 6).

For C–S–H with Ca/Si = 1.5, Moudrakovski *et al.*^28^ observed a clear left shoulder of the main peak which was not seen for Ca/Si = 1.2. This finding can be explained by the change in the silicate chain structure. The increase in Ca/Si is accompanied by a decrease in the MCL, therefore in an expected increase in the number of bridging Ca sites.^9,17^ Therefore, at Ca/Si = 1.5 a higher number of bridging Ca sites is expected than at Ca/Si = 1.2. While the spectrum is too broad to identify a clear chemical shift of this Ca site, it suggests the existence of a Ca site at higher frequencies, that can be assigned to the bridging Ca site.

The absence of the right shoulder of the main peak in experimental measurements of Moudrakovski *et al.*^28^ is consistent with the latest studies of the C–S–H surface structure. Zeta potential studies of C–S–H with Ca/Si < 1.5 synthesized from SiO_2_ and CaO are consistently negative,^77,78^ while those of C–S–H with Ca/Si > 1.5 synthesized with the drop-wise precipitation method are positive.^73,79^ As explained by Casar *et al.*,^9,18^ Ca^2+^ adsorption is necessary to reach high Ca/Si ratios which consequently causes positive zeta potentials. Therefore, at low Ca/Si ratios negative zeta potentials are expected due to the absence of Ca^2+^ adsorption sites on the surface. Since C–S–H synthesized from SiO_2_ and CaO is low in Ca/Si ratios, a negligible amount of surface Ca sites is expected, which is reflected by the absence of a right shoulder in the ^43^Ca NMR spectra of Moudrakovsi *et al.*^28^

### 3.3 Hydration of triclinic tricalcium silicate

In Fig. 1 it is seen that Ca exhibits a wide variety of chemical shifts, depending on its local environment, which arise from the different crystal structures. Since hydrated Portland cement is a mixture of different anhydrous (*e.g.* C_3_S, C_2_S, C_3_A) and hydrated phases (*e.g.* C–(A)–S–H, CH, C_6_A$_3_H_32_) it raises the question, if ^43^Ca NMR could be a useful technique for monitoring the hydration reaction of cement, where these phases coexist.

To provide additional insights in this matter, the hydration rate of tricalcium silicate (C_3_S) was computed by thermodynamic modelling at different hydration times (24 h, 92 h, and 463 h, equivalent to 43, 83 and 99% DoH, degree of hydration, respectively). A pure C_3_S paste was chosen as the model system considering that Moudrakovski *et al.*^28^ reported the DFT predicted ^43^Ca chemical shifts for this phase, which were in good agreement with the experimentally measured ^43^Ca NMR spectra of triclinic C_3_S.

**Fig. 7:**
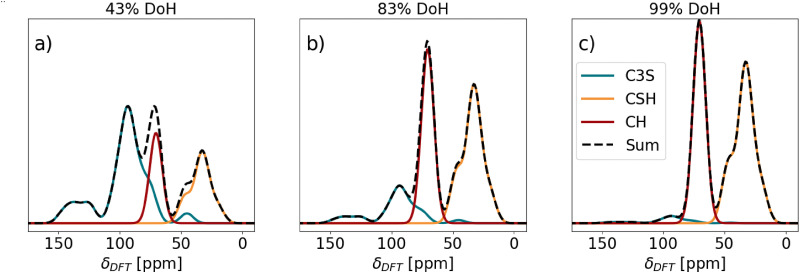
Predicted ^43^Ca NMR spectra of C_3_S pastes at different degrees of hydration (DoH): (a) 43%, (b) 83%, and (c) 99% of triclinic C_3_S. The C–S–H spectrum corresponds to the theoretical spectrum of Ca/Si = 1.75 in C–S–H (Fig. 6(c)).

Fig. 7 shows the predicted ^43^Ca NMR spectra at the different DoH of C_3_S studied. The here reported ^43^Ca NMR spectrum of C_3_S assumes an ^43^Ca enriched sample, therefore the resolution of this theoretical ^43^Ca NMR spectrum is much higher than of the experimentally measured one with natural abundance. At 43% DoH of C_3_S, the formation of C–S–H results in a separate peak at 33 ppm, while the formation of CH converts the right C_3_S shoulder into a separate peak. As the hydration reaction continues (83% DoH, Fig. 7(b)), the decrease in the C_3_S content is seen as a decrease in the main C_3_S peak at 93 ppm and its 150–120 ppm shoulder. Simultaneously, the C–S–H and CH peaks rise proportionally. The 70 ppm (CH) and 34 ppm (C–S–H) peaks become dominant. At 463 h (99% DoH, Fig. 7(c)), the C_3_S related resonances between 90 and 150 ppm almost completely disappear, and the C–S–H and CH peaks are the only remaining. Nevertheless, the spacing between the main C_3_S, CH and C–S–H lines in the ^43^Ca NMR spectra suggest that, in real experiments, deconvolution should be feasible to implement provided that the line shape is properly accounted for, enabling computation of the DoH of C_3_S even at later ages, along with a precise determination of CH content. As these parameters can also be quantitatively accessed by independent techniques (XRD and XRD/TGA, respectively), this could provide a pathway for the validation of results.

Moudrakovski *et al.*^28^ carried out ^43^Ca NMR spectroscopy on hydrated triclinic C_3_S. Since the authors studied the ^43^Ca NMR spectra under natural abundance of ^43^Ca the spectrum is very broad and noisy. However, the CH peak is clearly seen around 70 ppm. The absence of the left shoulder from the CH peak suggests that C_3_S fully reacted by the time of measurement. The spectrum has a wide range of broad signals from 20 to 70 ppm, and therefore the C–S–H peak cannot be distinguished from it. However, these theoretical study on triclinic C_3_S hydration (Fig. 7) shows that ^43^Ca NMR could be a useful tool in monitoring the cement reaction, provided that a better signal-to-noise ratio can be achieved.

## 4 Conclusion and outlook

The theoretical study of ^43^Ca NMR spectrum of C–S–H here presented has the potential to further help elucidate the C–S–H structure particularly in answering the question, of whether all synthetic C–S–H, from various precipitation methods, are the same. Due to the distinct chemical shift of C–S–H Ca sites it should be possible to compare the ^43^Ca NMR spectra of the samples from different synthesis methods, and obtain an estimation of the proportion of the Ca sites. With this a further step in understanding the interlayer and surface structure of C–S–H could be possible.

However, this can only be possible if experimental measurements are carefully combined with atomistic modeling. To ensure to have enough information to build a realistic C–S–H nanofoil model, the samples need to be simultaneously characterized by a wide variety of characterization methods. These methods should include:

• X-ray diffraction to prob the mainlayer ordering of C–S–H and get an idea of the layer spacing distance from the 002 reflection.

• ^29^Si NMR to determine the mean chain length (MCL) and consequently estimate the amount of bridging Ca sites.

• Inductive coupled plasma spectroscopy to determine the Ca/Si ratio of the sample.

• Specific surface area measurements from which the thickness of the C–S–H nanofoil can be estimated (which affects the proportion of Ca sites).

• Zeta potential measurement to estimate the surface charge and consequently the expected Ca coverage at the interface.

Additionally, extended X-ray absorption fine structure (EXAFS) investigation would be welcomed, due to its ability to measure the average Ca–O bond distance and coordination number.

Due to the Ca surface site exhibiting the lowest chemical shift, ^43^Ca NMR could potentially be useful in the study of the adsorption and desorption of Ca and Ca complexes at the C–S–H surface under various conditions.

It was shown here that ^43^Ca NMR could be successfully used to track the hydration reaction of cement or its individual phases. We believe particular interest could be in enriching a single phase of the clinker with ^43^Ca. With this it could be possible to obtain additional insights on the migration of Ca ions between the dissolving and precipitating phases, allowing distinction between (competing) Ca sources.

To answer the initial question: is there a future for ^43^Ca NMR in cement research? Yes, the above stated clearly shows that ^43^Ca NMR could be used in further elucidating the atomic-level structure of C–S–H as well as contribute to the understanding of its nucleation and growth and the general understanding of cement hydration. However, it is no easy task and it would require a combination of multiple characterization methods coupled with atomistic simulations that enable an effective interpretation and validation of experimental results. Due to the Ca nucleus characteristics, it is expected that promising results can only be expected from ^43^Ca enriched samples and high magnetic fields.

## Author contributions

Ziga Casar: conceptualization, methodology, software, investigation, writing – original draft. Davide Tisi: methodology, software, writing – review & editing, supervision. Samuel J. Page: writing – review & editing, supervision. H. Chris Greenwell: resources, writing – review & editing, supervision. Franco Zunino: conceptualization, methodology, investigation, writing – original draft, project administration.

## Data availability

The data supporting this article have been included as part of the ESI.†

## Conflicts of interest

There are no conflicts of interest to declare.

## Supplementary Material

CP-027-D5CP00491H-s001
